# A Smart Shoe System for Gait Analysis and Remote Monitoring in Parkinson’s Disease—A Validation Study

**DOI:** 10.3390/s26144446

**Published:** 2026-07-13

**Authors:** Shanshika Maddumage Dona, Karen Sullivan, Alexander Lehn, Nadeesha Kalyani, Danielle Prins, Graham Kerr

**Affiliations:** 1 School of Exercise and Nutrition Sciences, Faculty of Health, Queensland University of Technology, Brisbane, QLD 4059, Australia; shanshika.dona@hdr.qut.edu.au; 2 School of Psychology and Counselling, Faculty of Health, Queensland University of Technology, Brisbane, QLD 4059, Australia; karen.sullivan@qut.edu.au; 3 Department of Neurology, Princess Alexandra Hospital, Brisbane, QLD 4102, Australia; alexander.lehn@health.qld.gov.au; 4 School of Biomedical Sciences, Faculty of Health, Queensland University of Technology, Brisbane, QLD 4059, Australia; 5 School of Health, Psychological and Medical Sciences, University of Southern Queensland, Toowoomba, QLD 4350, Australia; nadeesha.kalyani@unisq.edu.au; 6 School of Clinical Sciences, Faculty of Health, Queensland University of Technology, Brisbane, QLD 4059, Australia; danielle.prins@hdr.qut.edu.au

**Keywords:** wearable sensors, motion capture, agreement analysis, spatiotemporal gait parameters, gait monitoring, haptic feedback, vibrotactile cueing, concurrent validity

## Abstract

**Highlights:**

**What are the main findings?**

- The NUSHU smart shoe system demonstrated good agreement with 3D motion capture system for measuring key spatiotemporal gait parameters in people with Parkinson’s disease and age- and gender-matched older adults.
- Acceptable agreement was mainly found for stride length, stride time, and velocity, while double-support time still requires further validation.

**What are the implications of the main findings?**

- The NUSHU system provides a valid and reliable wearable approach for quantifying gait parameters across both clinical and healthy older adult populations.
- This system shows potential as a wearable option for gait assessment, although further validation in free-living or real-world settings is needed to confirm its utility outside the laboratory.

**Abstract:**

Continuous gait monitoring is important in Parkinson’s Disease (PD) as gait deteriorates with disease progression and responses to treatment vary over time. Wearable sensor systems offer a practical alternative to laboratory-based gait assessment for real-world monitoring. This study aimed to (1) validate spatiotemporal gait parameters derived from the NUSHU smart shoe system against the Vicon motion capture system in healthy older adults and individuals with PD, and (2) assess whether measurement agreement was maintained during active vibrotactile feedback delivery. Thirty-two participants (17 with mild to moderate PD and 15 age- and gender-matched healthy older adults; mean age 67 ± 7.4 years) completed overground walking trials under no vibration, stance phase vibration, and swing phase vibration conditions. A total of 4790 strides were analysed using linear mixed effects models. Overall agreement was good for stride length (ICC = 0.894, CCC = 0.926), stride time (ICC = 0.853, CCC = 0.954), and velocity (ICC = 0.885, CCC = 0.940), with moderate agreement for double support time (ICC = 0.752, CCC = 0.477). Agreement remained consistent across vibration conditions in both groups. The NUSHU system demonstrates acceptable validity for stride length, stride time, and velocity under both measurement and active feedback modes.

## 1. Introduction

Gait impairment is a hallmark feature of Parkinson’s disease (PD) and contributes to reduced mobility, increased fall risk, and loss of independence [1]. These impairments in PD are characterised by reduced step length, slower walking velocity, increased gait variability, and prolonged double support time, with features worsening as the disease progresses from early to advanced stages [2]. Prolonged double support in particular reflects a compensatory strategy to maintain postural control and reduce fall risk [3]. Quantitative gait analysis provides objective measures of these spatiotemporal gait characteristics and is widely used to monitor disease progression and evaluate treatment outcomes in clinical practice [4]. Accurate identification of gait abnormalities at an early stage is therefore essential in PD for optimising clinical management and rehabilitation planning [5].

Marker-based 3D motion-capture systems are currently the gold standard for gait analysis [6]; however, data acquisition using motion capture is time-consuming, expensive, and requires specialised personnel, environments, and equipment [7]. Consequently, wearable sensor technologies have gained increasing attention as a practical alternative for gait assessment, offering the potential for more accessible and real-world gait monitoring [7]. Moreover, real-world gait monitoring using wearable sensors enables clinicians to closely track patients’ progress, evaluate treatment effectiveness, and optimise management strategies, potentially reducing the need for frequent in-person clinic visits [8].

Even though wearable sensor technologies provide an opportunity for objective and continuous gait monitoring outside laboratory environments [9,10] several practical limitations remain. Some systems require multiple sensors, making them impractical for everyday use and increasing user burden. Adherence may also be challenging, particularly for individuals with cognitive impairment who may require assistance to reattach sensors after accidental removal [11]. In addition, the clinical utility of wearable-derived gait measures is influenced by their variable measurement accuracy and sensitivity [12]. Therefore, rigorous validation against established reference systems is essential to ensure that wearable sensor systems provide accurate, reliable, and clinically meaningful gait assessments [12]. These limitations highlight the need for simple, practical and user-friendly wearable solutions that can support valid gait monitoring in real-world settings.

Accurate measurement of gait parameters is essential for the clinical use of wearable sensors, as these systems must be sensitive enough to detect changes that exceed minimal clinically important difference (MCID) thresholds [13]. MCID represents the smallest change in an outcome that is perceived as meaningful by patients and may influence clinical decision-making [14]. Therefore, ensuring the validity and precision of wearable-derived gait measures is essential for their accurate clinical interpretation and use in treatment monitoring.

Previous validation studies have evaluated in-shoe wearable sensor systems for measuring spatiotemporal gait parameters in people with PD by comparing their outputs with laboratory-based reference methods, including motion-capture systems [15,16,17] and instrumented walkways [18,19]. The technologies investigated have ranged from shoe-mounted inertial measurement unit (IMU) systems and multi-sensor wearable platforms to instrumented insoles. Despite these advances, several limitations remain. Some studies have relied on averaged gait outcomes rather than step-by-step analysis [18], included small sample sizes [17], lacked appropriate control groups [19], or assessed gait primarily during treadmill walking rather than overground conditions [15]. These limitations restrict the generalisability of existing findings and highlight the need for further validation of practical in-shoe wearable sensor systems during overground walking, specifically evaluating stride-level agreement in both people with PD and healthy older adults, and assessing measurement accuracy under both vibration and no-vibration conditions during active vibrotactile feedback delivery.

The NUSHU system (Magnes AG, Zurich, Switzerland) used in this study is an in-shoe wearable sensor system developed for spatiotemporal gait assessment [20]. It consists of a pair of shoes embedded with IMUs controlled via a dedicated mobile application (iOS). In addition to gait measurement, the system delivers programmable vibrotactile cueing timed to specific phases of the gait cycle, such as stance or swing, via an embedded vibration motor in the shoe sole. External cues are intended to shift habitual, internally driven motor control toward more goal-directed, externally driven control, drawing attention to the act of walking [21]. Vibrotactile cueing is one such approach that has emerged as a promising strategy for improving spatiotemporal gait parameters, including stride length, walking speed and cadence, in people with PD [22,23,24].

A preliminary validation study [20] compared NUSHU data with the Vicon 3D motion capture system (Vicon Oxford Metrics Ltd., Oxford, UK) and reported promising findings. However, that study included only four healthy participants, limiting the generalisability of the results to clinical populations. Importantly, the system has previously been validated only under non-vibration conditions. As vibrotactile cueing is generated by vibration motors embedded in the shoes, the resulting external mechanical disturbances may interfere with IMU measurements, since accelerometers and gyroscopes are inherently susceptible to noise from such disturbances [25]. Therefore, it is important to test the robustness of NUSHU gait parameters derived from proprietary algorithms by assessing whether their accuracy differs between vibration and non-vibration conditions, thereby determining whether sufficient compensation for these effects has been achieved.

Therefore, the primary purpose of this study was to evaluate the clinical feasibility, accuracy, and validity of the NUSHU system within a clinical environment for measuring spatiotemporal gait parameters in both healthy participants and individuals with PD, against the gold-standard Vicon motion-capture system. A secondary aim was to evaluate performance in no-vibration versus vibration modes. We hypothesised that the spatiotemporal gait parameters derived from the NUSHU smart shoes, stride length, stride time, velocity, and double support time, would exhibit good agreement with those obtained from the Vicon motion capture system, and that this level of agreement would remain consistent regardless of whether the vibration mode was enabled or disabled.

## 2. Materials and Methods

### 2.1. Study Design, Setting and Participants

An analytical cross-sectional study design was used in the current study. The study was conducted in the gait laboratory at Queensland University of Technology in Brisbane, Australia. The common inclusion criteria for both healthy participants and people with PD were aged between 40 and 85 years, able to walk independently without an assistive device and able to read, write and understand English. A minimum score of 22 points on the Montreal Cognitive Assessment (MoCA) was required to ensure that people with PD could provide informed consent for research [26]. For people with PD, additional inclusion criteria were a diagnosis of idiopathic PD confirmed by a neurologist and a Hoehn and Yahr stage I–III, indicating mild to moderate disease with the ability to ambulate independently. Individuals with confounding medical, neurological, musculoskeletal, cardiovascular, or respiratory abnormalities, or who had undergone deep-brain stimulation, were excluded. Participants with PD were assessed in their self-reported ON medication state. Participants were instructed to take their usual antiparkinsonian medication at least one hour before testing, and the time since the last medication dose was recorded during the MDS-UPDRS assessment. Testing was paused if participants required additional medication during testing until ON medication status was retained.

Healthy older adults were matched 1:1 with participants with PD by sex and age (±1 year) at recruitment. However, the final analysed sample was no longer strictly 1:1 matched after technical exclusions. The study procedures were reviewed and approved by the University Human Research Ethics Committee (UHREC) of the Queensland University of Technology (Approval number: 7473). All participants signed an informed consent prior to participation in the study.

### 2.2. Sample Size

A priori sample size calculation was performed based on the intraclass correlation coefficient (ICC) hypothesis testing [27,28] to determine the number of participants required to assess agreement between the NUSHU smart shoe system and the reference motion capture system. Assuming a minimum acceptable ICC of 0.50 and an expected ICC of 0.80, with a two-tailed significance level of 0.05 and 80% power, a minimum sample size of 28 participants was required. Allowing for an anticipated 10% data loss, the required sample size was increased to 32 participants. The sample size calculation was based on the number of participants, consistent with the participant-level unit of inference used in all statistical analyses.

### 2.3. Instrumentation

#### 2.3.1. Magnes NUSHU Smart Shoes

The study used five pairs of Magnes NUSHU (Magnes AG, Zurich, Switzerland) (European sizes 39–47), which is a wearable IMU-based smart shoe with real-time haptic feedback capability. The hardware platform has been described in detail previously [20,29]. Briefly, each shoe integrates a custom sensor unit embedded within the posterior portion of the outsole, housing a tri-axis accelerometer and tri-axis gyroscope (LSM6DSM, STMicroelectronics, Geneva, Switzerland; 16-bit resolution) configured to ±8 g and ±2000°/s respectively, and a tri-axis magnetometer (LSM303AGR, STMicroelectronics, Geneva, Switzerland), alongside a 32-bit microcontroller, Bluetooth Low Energy communication module, and a vibration motor for provision of haptic feedback.

Data were acquired at a sampling frequency of 100 Hz [20]. No additional calibration was performed beyond the manufacturer’s on-board automatic calibration routines. Device configuration, data streaming, and management were performed via the NUSHU mobile application (iOS application version 18.7.9; firmware version 1.5.1). Gait event detection and spatiotemporal parameter extraction were performed using the NUSHU cloud-based processing platform. The specific gait detection algorithms are proprietary to Magnes AG, and key features of the algorithm have been described previously [29].

The NUSHU system delivers suprathreshold haptic feedback via embedded vibration motors located within the shoe sole, with vibration timing configurable to target either the stance or swing phase of gait for each foot. The vibration motor operated at 125 Hz. Vibration conditions are selected via the NUSHU mobile application prior to data acquisition. Gait phase transitions are detected in real time using the onboard inertial sensors, with vibration delivered independently for each shoe and triggered automatically by the on-board gait event detection algorithm.

#### 2.3.2. Vicon Motion Capture System

Gait data were collected using a 12-camera Vicon T40 three-dimensional motion analysis system (Vicon MX, Oxford Metrics, Oxford, UK) with a sampling rate of 100 Hz. Data acquisition and processing were performed in Vicon Nexus software, version 2.15.

#### 2.3.3. Vicon Blue Trident Inertial Measurement Unit

Two Vicon Blue Trident inertial measurement units (Vicon Motion Systems Ltd., Oxford, UK) were secured laterally on each shoe, aligned with the inbuilt NUSHU IMUs. The IMUs recorded tri-axial acceleration, angular velocity, and magnetometer data at a sampling frequency of 1125 Hz. These IMUs were connected to the Vicon Nexus system and used solely for synchronisation.

#### 2.3.4. The ProtoKinetics Zeno™ Walkway Gait Analysis System

The ProtoKinetics Zeno™ Walkway Gait Analysis System (Z744) (ProtoKinetics, Havertown, PA, USA) was used to ensure synchronisation between different systems. However, Zeno Walkway data are not reported as the present analysis focuses on validation against the Vicon system.

### 2.4. Experimental Protocol

**Figure 1:**
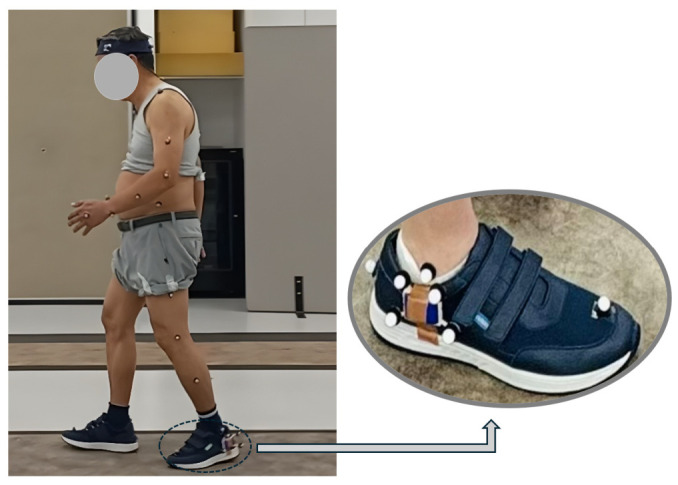
Participant performing a walking trial with reflective markers and IMUs attached. The inset depicts the placement of the Vicon IMU and the attached marker cluster.

Thirty-nine reflective markers (14 mm diameter) were bilaterally attached to specific anatomical landmarks using double-sided tape, following the Vicon full-body Plug-in Gait model to enable three-dimensional analysis of joint kinematics during walking. Two IMUs were attached laterally to the shoes, in line with the NUSHU IMU just below the lateral malleolus, and had a marker cluster affixed to them (Figure 1).

At the beginning of each walking trial, the NUSHU system delivered two distinct vibrations. These initial vibrations were detected by the shoe-mounted IMUs and served as reference events to align the NUSHU and Vicon data streams. The Zeno walkway was connected to Vicon via a sync-out trigger; thus, when data recording started in Zeno, it automatically triggered Vicon to begin capturing data. Together, these procedures ensured synchronisation across the NUSHU, Zeno, and Vicon systems.

Participants began each walking trial at one end of the gait lab and walked to the other end. To allow for acceleration, they walked 1.5 m before stepping onto the Zeno walkway (8.2 m long). After stepping off the walkway, they continued to walk for an additional 1.5 m to allow for deceleration. The 1.5 m buffer zones ensured participants had reached steady-state walking before entering the Vicon capture volume, providing enough complete strides for NUSHU-Vicon validation. It is acknowledged that, in real-world use, the NUSHU system captures all phases of walking, including acceleration and deceleration, which fell outside the Vicon capture volume and were therefore excluded from the analysis.

Each participant completed 20 walking trials: (1) no vibration (baseline 1), (2) vibration during the stance phase, (3) no vibration (baseline 2), (4) vibration during the swing phase, and (5) no vibration (baseline 3). Four trials were performed under each condition. Prior to each walking trial, the experimenter selected the vibration condition via the NUSHU application, which remained active throughout that trial. Vibration intensity was standardised across all participants by setting the NUSHU application to 100% (maximum output), which corresponds to the manufacturer-defined maximum stimulation level; vibration amplitude was not directly measured in physical units. During stance-phase vibration trials, stimulation was delivered from heel contact until toe-off. During swing-phase vibration trials, stimulation was delivered from toe-off until the subsequent heel contact. Given that the primary aim of this study was to evaluate the concurrent validity of the NUSHU system relative to the reference system, condition order was kept consistent across participants rather than randomised. To minimise potential carry-over effects from vibration exposure, each vibration condition was followed by a no-vibration baseline condition before the next vibration condition was introduced. Adequate rest periods were also provided between trials to reduce the likelihood of fatigue-related changes in gait. Whilst vibrotactile cueing has been shown to influence proprioceptive feedback and gait parameters in some contexts, any such effect on gait would be expected to affect NUSHU and Vicon measurements equally, given simultaneous capture, and would therefore not bias the concurrent validity assessment.

### 2.5. Outcome Measures

The spatiotemporal parameters considered for the analysis were as follows:

- Stride Length (m): The distance between the initial contact (heel strike was considered as the initial contact) of one foot and the successive initial contact of the same foot.
- Stride time (s): The time duration between the heel strike of one foot to the following heel strike of the same foot.
- Stride velocity (m/s): The ratio of stride length to stride time.
- Double support time (s): The sum of all periods when both feet are in contact with the ground during the stance phase.

### 2.6. Data Processing

#### 2.6.1. Vicon Motion Capture Data

All marker trajectories were reconstructed and labelled using Vicon Nexus software (version 2.15) with the Plug-in Gait full-body pipeline. After model fitting, gait events (heel strike and toe-off) were manually identified by visual inspection of the heel and toe marker trajectories in Vicon Nexus. The minimum vertical positions of the heel and toe markers were used to identify heel strike and toe-off, respectively. Spatiotemporal gait parameters were then calculated using the Vicon Nexus “Calculate Gait Cycle Parameters” pipeline. Individual gait cycle data were exported via the “Run ProCalc” pipeline, and additional temporal parameters were derived from the exported CSV files containing gait event data. Vicon gait event identification was performed using Vicon data only and was completed prior to receiving and comparing with NUSHU-derived gait parameters.

#### 2.6.2. NUSHU Smart Shoe Data

NUSHU data were processed using Magnes’ cloud-based analytics platform to obtain spatiotemporal gait parameters. The processed gait parameter data were subsequently downloaded from the NUSHU Toolbox (version 1.4.0) for comparison with the reference systems.

#### 2.6.3. Device Synchronisation

At the start of each walking trial, the NUSHU smart shoes generated two vibration pulses (200 ms duration) using the embedded vibration motors. The motor-driving binary signal (ON/OFF) was recorded as part of the NUSHU dataset and sampled alongside linear acceleration and angular velocity signals at 100 Hz. These vibration events were also detected by the linear accelerometers of the Vicon IMUs. The following synchronisation strategies were implemented to estimate the temporal offset (Δt) between the NUSHU and Vicon time bases for each side. One strategy was based on the cross-correlation between the NUSHU and Vicon IMUs. The other strategy was based on the signal energy detected by the IMUs. Three candidate synchronisation offsets were obtained for each side and were manually reviewed by visually inspecting the effects of each candidate on the raw signals. This approach guarded against residual artefacts specific to any single method.

After selecting the appropriate synchronisation offset for each side, this was applied to all NUSHU timestamps prior to further processing. Following temporal alignment, the first heel strike event identified by both systems and showing close temporal agreement was defined as time zero for that trial. A detailed description of the synchronisation procedure is provided in Supplementary Material.

### 2.7. Statistical Analysis

Data analysis was performed using R (version 4.6.0, R Core Team, Vienna, Austria) and IBM SPSS (version 31, IBM Corp., Armonk, NY, USA). Demographic characteristics and descriptive statistics of gait parameters were calculated as means ± standard deviations (SD). As strides are nested within participants and trials, all primary agreement statistics were derived from linear mixed-effects models (LME) using the lme4 package in R, with device (Vicon vs. NUSHU) as a fixed effect and random intercepts for participant and trial to account for the repeated-measures structure. Intraclass correlation coefficients were derived from the variance components of the mixed effects model with 95% confidence intervals. ICC values below 0.50 were considered poor, 0.50–0.75 moderate, 0.75–0.90 good, and above 0.90 excellent [30]. Concordance correlation coefficients (CCC) were calculated using the DescTools package (version 0.99.60) in R as a descriptive index of absolute agreement between devices. Systematic bias and limits of agreement (LoA) between devices were estimated from a linear mixed-effect model fitted to the per-stride differences, with random intercepts for participant and trial to account for repeated measures. Bias was defined as the model fixed intercept, and LoA as bias ± 1.96 × the total standard deviation derived from all model variance components. Bland-Altman plots were constructed to visualise agreement between systems [31]. Mean absolute error (MAE) was reported as a clinically interpretable measure of average measurement error. To assess consistency of agreement across groups and vibration conditions, ICC and CCC were computed separately for PD and healthy participants within each vibration category. For visualisation purposes, scatter plots of NUSHU against Vicon measurements were produced using simple linear regression with Pearson correlation coefficients reported. This approach was adopted because it captures agreement across the full range of gait values, while formal agreement statistics (ICC, bias, and limits of agreement) were derived from linear mixed-effects models as described above. An a priori clinical acceptability threshold was defined for gait velocity based on its established MCID of 0.082 m/s in PD [32]. A conservative clinical benchmark of Δ = 0.041 m/s (approximately half the MCID) was used to interpret whether the observed bias was clinically meaningful. For the remaining gait parameters, robust MCID values do not appear to be well established in the literature. Therefore, agreement for these parameters was interpreted descriptively based on bias and limits of agreement.

## 3. Results

### 3.1. Participant Characteristics

A total of 34 participants were recruited for the study. Data from two older adults were excluded from the final analysis due to technical issues during data capture. The final sample, therefore, comprised 32 participants (17 with PD and 15 healthy older adults). Demographic and clinical characteristics of these 32 participants are shown in Table 1. Participants with PD had mild-to-moderate disease, with an average MDS-UPDRS III of 16.41 ± 9.30, and an average Hoehn and Yahr score of 2.06 ± 1.29.

### 3.2. Gait Data Overview

A total of 4790 strides were analysed across 32 participants, including 2712 strides from participants with PD. Across all participants, 2877 strides were collected without vibration, 958 with stance-phase vibration and 955 with swing-phase vibration. Within the PD group, 1604 strides were recorded without vibration, 547 with stance-phase vibration and 561 with swing vibration. In the healthy control group, 1273 strides were collected with no vibration, 411 with stance-phase vibration, and 394 with swing-phase vibration.

### 3.3. Agreement Between the NUSHU Smart Shoes and the Vicon System

Table 2 presents the descriptive statistics and ICCs for gait parameters obtained from the NUSHU smart shoe system and the Vicon motion capture system. The results include pooled data from all participants across both groups and all experimental conditions.

Good reliability was observed between the NUSHU and Vicon systems for stride length, stride time, and stride velocity (ICC range: 0.792 to 0.940), whereas double support time showed moderate reliability (ICC range: 0.661 to 0.843).

### 3.4. Correlation Analysis

**Figure 2:**
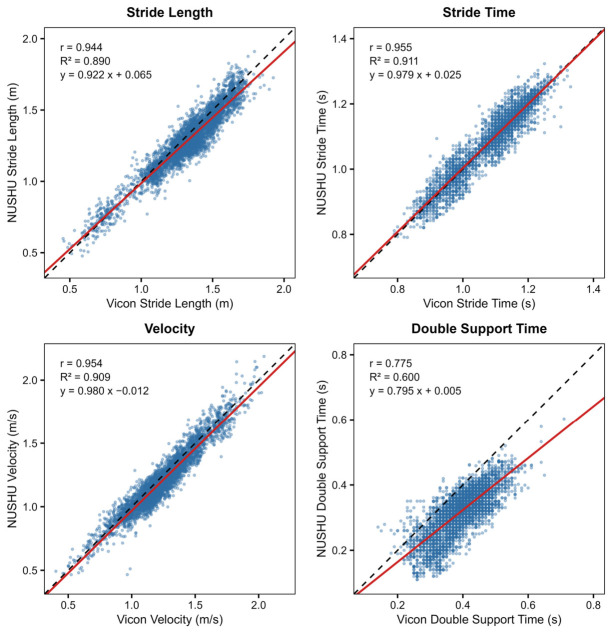
Scatter plots illustrating the relationship between Vicon and NUSHU measurements for four gait parameters. The red line represents the linear regression line, and the black dashed line represents the line of perfect agreement. r = Pearson correlation coefficient.

Strong positive correlation between NUSHU and Vicon for stride length (r = 0.944, y = 0.922x + 0.065), stride time (r = 0.955, y = 0.979x + 0.025) and velocity (r = 0.954, y = 0.980x − 0.012), with regression lines closely approximating the line of perfect agreement. Double support time showed a moderate correlation (r = 0.775, y = 0.795x + 0.005) with increasing deviation from the line of perfect agreement at longer durations, suggesting that NUSHU underestimates double support time particularly during slower gait (Figure 2).

### 3.5. Bland–Altman Analysis

**Figure 3:**
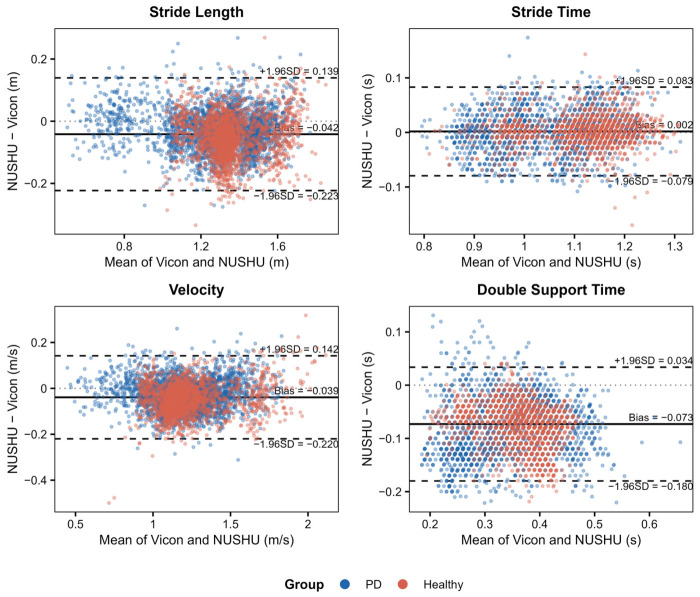
Bland–Altman plots illustrating agreement between Vicon and NUSHU measurements for four gait parameters. The x-axis represents the mean of the two measurements, and the y-axis represents the difference (NUSHU minus Vicon). Blue circles represent PD participants and red circles represent healthy older adults. The solid black line represents the mean bias, and the dashed black lines represent the 95% limits of agreement.

Agreement between the two measurement systems is illustrated in the Bland–Altman plots (Figure 3). Bland–Altman analysis showed small mean biases between the two systems for stride length (bias = −0.042 m, 95% LoA: −0.223 to 0.139 m), stride time (bias = 0.002 s, 95% LoA: −0.079 to 0.083 s), and velocity (bias = −0.039 m/s, 95% LoA: −0.220 to 0.142 m/s), indicating minimal systematic differences between the two systems. However, the 95% limits of agreement indicate variability at the individual level. The results for velocity should therefore be interpreted with caution. Double support time showed a larger mean bias (−0.073 s, 95% LoA: −0.180 to 0.034 s), suggesting that while group-level estimates may be acceptable, the wider limits of agreement indicate greater variability at the individual stride level and should be interpreted with caution in clinical applications requiring precise individual-level measurement.

### 3.6. Agreement Between NUSHU and Vicon Across Participant Groups (PD vs. Healthy) and Vibration Conditions

To evaluate whether vibrotactile stimulation affected the accuracy of NUSHU gait measurements, agreement between NUSHU and Vicon was examined across three conditions: no vibration, stance-phase vibration, and swing-phase vibration (Table 3). In the PD group, ICC values remained consistently comparable across conditions for all gait parameters (Table 3). Stride length ICC was 0.899, 0.944, and 0.932; stride time 0.871, 0.860, and 0.904; velocity 0.885, 0.922, and 0.917; and double support time 0.764, 0.775, and 0.782, for no vibration, stance-phase vibration, and swing-phase vibration, respectively. A similar pattern was observed in the healthy older adults’ group, where ICC values across the same conditions were 0.875, 0.876, and 0.895 for stride length; 0.814, 0.834, and 0.866 for stride time; 0.880, 0.905, and 0.922 for velocity; and 0.735, 0.762, and 0.796 for double support time. CCC values and MAE followed the same trend across both groups, with no systematic deterioration in agreement associated with either vibration condition.

## 4. Discussion

The NUSHU smart shoe system showed acceptable agreement with the gold-standard motion-capture system for stride length, stride time, and velocity in people with PD and in age- and gender-matched older adults. There was good agreement for stride length, stride velocity, and stride time, but moderate agreement for double support time. This agreement was maintained regardless of whether vibration was applied during the swing or stance phases of gait. These results extend a previous validation study [20] to encompass both older people and people with PD. A validated wearable gait-monitoring system potentially enables continuous gait assessment, which in turn could yield significant clinical and patient benefits [33,34,35,36].

The NUSHU system closely aligns with the Vicon motion capture system for stride length, stride time and velocity, with higher ICC values. The lower ICC values observed for double-support time may be attributable to limitations in temporal synchronisation between the left and right sensors of the NUSHU system. Because the system’s algorithm processes left and right-foot data independently, double-support time is derived by merging these two data streams under the assumption of precise bilateral synchronisation. Any minor misalignment between sides could therefore propagate into the double-support time calculation, reducing measurement reliability. Moreover, unlike stride time or stride length, which are derived from a single pair of repeated events on the same limb, double-support time is calculated from four discrete gait events across both limbs. Therefore, any minor misalignment between the left and right sensor clocks would propagate directly into the double-support time calculation, even if each individual foot’s gait events were detected accurately.

### 4.1. Agreement Between NUSHU and Vicon Across Participant Groups (PD vs. Healthy)

The agreement between the NUSHU smart shoes and the Vicon motion capture system was higher in participants with PD than in healthy older adults, as reflected by higher ICC values in the PD group. One possible explanation for this pattern may be that people with PD may exhibit greater gait variability across a wide range of spatiotemporal parameters compared to healthy older adults, which could influence ICC estimates. As ICC is sensitive to between-subject variability, a broader distribution of gait parameters increases the proportion of true variance relative to measurement error, resulting in higher ICC estimates [37,38]. In contrast, healthy participants typically exhibit more consistent gait patterns and a restricted range of values, which can reduce ICC estimates independently of device accuracy. However, this difference in ICC values between groups should be interpreted with caution.

### 4.2. Agreement Between NUSHU and Vicon Across Different Vibration Conditions

The condition-based subgroup analysis showed that ICC values were consistent regardless of whether the vibration mode was on or off. These findings suggest that the vibration motor embedded in the shoe sole does not affect the performance of the NUSHU gait event detection algorithm.

### 4.3. Comparison with Previous Wearable Sensor Validation Studies in Parkinson’s Disease

Our findings align with prior studies comparing wearables and reference systems that have reported excellent agreements (ICC values > 0.9) for measures such as step/stride length [15,17,18,19], step/stride time [15,17,18,19], and gait velocity [17,18,19]. Similar to earlier work [18,19], double support time showed only poor agreement in our study, which may be attributable to its greater sensitivity to subtle gait timing variability, particularly in individuals with PD. Importantly, the current study extends previous research by demonstrating step-by-step agreement during overground walking in a cohort that included both healthy older adults and individuals with PD.

### 4.4. Minimal Clinically Important Difference (MCID) Values and Mean Differences Between NUSHU and Vicon

Among the evaluated gait parameters, gait velocity is particularly clinically relevant because it integrates multiple aspects of gait performance and has been shown to predict functional status, health outcomes, and survival in older adults [39,40], while also serving as a commonly reported outcome measure in PD rehabilitation studies and in the clinical assessment of disease severity [2]. Moreover, gait velocity has the most robustly established MCID in PD (0.082 m/s) [32], whereas equivalent threshold values for stride length and double support time remain less consistently defined in the literature. Therefore, gait velocity was selected for assessing whether the systematic error observed between NUSHU and Vicon falls within a clinically meaningful range.

Accordingly, a conservative clinical benchmark of Δ = 0.041 m/s, deliberately below the MCID was set for the present study. The mean difference in gait velocity between the NUSHU smart shoe system and the motion capture system observed in the current study (−0.039 m/s) remained within this benchmark, suggesting that the observed systematic error is below the threshold for clinically meaningful change. However, the 95% limits of agreement (0.220 to 0.142 m/s) exceeded the MCID, indicating that individual-level measurements should be interpreted with caution.

### 4.5. Strengths and Limitations

The current study has several strengths and limitations. A key strength was the inclusion of both people with PD and age and gender-matched healthy older adults. The sample included both male and female participants, allowing for gender-related variability in gait. Compared with previous validation studies of wearable sensors, the study analysed many strides across participants and conditions. To account for the repeated-measures structure of the data, all agreement statistics were derived from linear mixed-effects models with random intercepts for participant and trial, ensuring that the nested structure of strides within participants was appropriately addressed. This enabled a robust comparative analysis between systems across a wide range of gait patterns, rather than relying solely on mean values.

One limitation of this study was that data collection was restricted to short walking distances (8 m) due to the Vicon capture volume. Consequently, the natural variability of gait typically observed in real-world or outdoor environments may not have been fully captured, even though the NUSHU system is designed for continuous real-world gait monitoring. A second limitation is that the analysis was restricted to straight-line walking; other functional tasks, such as turning or walking on uneven surfaces, were not evaluated. Third, gait was assessed only at a self-selected walking speed, without examining performance under slow or fast walking conditions.

### 4.6. Future Directions

Future studies should evaluate the NUSHU smart shoe system in free-living environments to capture gait variability over longer walking distances and during daily activities, including turning, walking on uneven surfaces, and at different walking speeds. Longitudinal monitoring using stride-to-stride analysis may further elucidate gait changes associated with disease progression, treatment response, and fall risk in PD. Such work could inform future research on integrating wearable gait monitoring into routine clinical practice to improve patient management, including continuous gait monitoring between scheduled visits, helping clinicians detect early signs of disease progression or medication wearing-off effects, and supporting more timely treatment adjustments.

## 5. Conclusions

The NUSHU smart shoe system shows acceptable agreement with a gold-standard motion-capture system for stride length, stride time and velocity during short-distance overground laboratory walking. The agreement for double support time was comparatively low and requires further development to synchronise the input from both shoes. Importantly, measurement accuracy was maintained across no-vibration and vibration conditions, supporting the concurrent use of the system for gait monitoring during vibrotactile cue delivery. This is a relevant finding in the context of PD, where vibrotactile cueing has been explored as a strategy to address gait disturbances. The present findings suggest that measurement accuracy of the NUSHU system was not adversely affected by concurrent vibrotactile cue delivery. Given that the present validation was conducted under standardised laboratory conditions, these findings support the use of the NUSHU system in similar testing environments, such as laboratories or clinics, for measuring gait parameters other than double support time. Its potential application for continuous monitoring in real-world, free-living environments remains to be established.

## Figures and Tables

**Table 1:** Demographic and clinical characteristics of participants (Mean and SD).

	Participants with PD (*n* = 17)	Older Adults (*n* = 15)
Age (Years)	67.1 ± 7.91	65.53 ± 7.06
Gender (M/F)	12 M/5 F	10 M/5 F
Height (cm)	172. 41 ± 9.08	160.51 ± 25.11
Weight (kg)	76.17 ± 12.11	80.90 ± 30.70
MoCA	28.17 ± 2.24	26.73 ± 1.53
Disease duration	5.82 ± 4.12	NA
H & Y score	2.06 ± 1.29	NA
MDS-UPDRS I score	10.76 ± 4.40	NA
MDS-UPDRS II score	11.59 ± 8.68	NA
MDS-UPDRS III score	16.41 ± 9.30	NA
MDS-UPDRS IV score	2.94 ± 2.19	NA
MDS-UPDRS Total score	41.71 ± 18.82	NA

MoCA = Montreal Cognitive Assessment, H & Y = Hoehn and Yahr, MDS-UPDRS = Movement Disorder Society-Unified Parkinson’s Disease Rating Scale, NA = Not applicable.

**Table 2:** Descriptive statistics and agreement metrics between NUSHU and Vicon gait parameters including intraclass correlation coefficient (ICC), concordance correlation coefficient (CCC), mean difference, and mean absolute error (MAE) across all participants and conditions.

Parameter	Mean ± SD Vicon	Mean ± SD NUSHU	Mean Difference	ICC	95% CI of ICC		CCC	MAE
					Lower Bound	Upper Bound		
Stride length (m)	1.336 ± 0.205	1.296 ± 0.200	−0.042	0.894	0.847	0.940	0.926	0.063
Stride time (s)	1.084 ± 0.096	1.086 ± 0.098	0.002	0.853	0.792	0.914	0.954	0.020
Velocity (m/s)	1.239 ± 0.218	1.202 ± 0.223	−0.039	0.885	0.836	0.934	0.940	0.061
Double support time (s)	0.392 ± 0.066	0.317 ± 0.068	−0.073	0.752	0.661	0.843	0.477	0.077

SD = Standard deviation, ICC = Intraclass correlation coefficient, CI = Confidence interval, CCC = Concordance correlation coefficient, MAE = Mean absolute error.

**Table 3:** Descriptive statistics, intraclass correlation coefficients, concordance correlation coefficients, mean differences, and mean absolute errors comparing NUSHU and Vicon gait parameters between the two groups across different vibration conditions.

PD Group	No Vibration (*N* = 1604 Strides)						Stance Vibration (*N* = 547 Strides)						Swing Vibration (*N* = 561 Strides)					
	Mean ± SD Vicon	Mean ± SD NUSHU	Mean Difference	ICC (95% CI)	CCC	MAE	Mean ± SD Vicon	Mean ± SD NUSHU	Mean Difference	ICC (95% CI)	CCC	MAE	Mean ± SD Vicon	Mean ± SD NUSHU	Mean Difference	ICC (95% CI)	CCC	MAE
Stride Length (m)	1.312 ± 0.208	1.281 ± 0.204	−0.031	0.899 (0.839, 0.959)	0.941	0.056	1.253 ± 0.273	1.229 ± 0.264	−0.024	0.944 (0.909, 0.979)	0.967	0.054	1.300 ± 0.239	1.268 ± 0.232	−0.032	0.932 (0.891, 0.974)	0.950	0.06
Stride time (s)	1.063 ± 0.097	1.065 ± 0.100	0.001	0.871 (0.797, 0.945)	0.944	0.023	1.057 ± 0.094	1.059 ± 0.098	0.002	0.860 (0.781, 0.940)	0.942	0.024	1.062 ± 0.109	1.064 ± 0.112	0.002	0.904 (0.847, 0.961)	0.960	0.022
Velocity (m/s)	1.241 ± 0.215	1.211 ± 0.219	−0.03	0.885 (0.818, 0.952)	0.945	0.057	1.187 ± 0.257	1.164 ± 0.253	−0.023	0.922 (0.875, 0.969)	0.965	0.055	1.226 ± 0.221	1.197 ± 0.228	−0.03	0.917 (0.867, 0.967)	0.941	0.062
Double support time (s)	0.386 ± 0.070	0.309 ± 0.079	−0.077	0.764 (0.644, 0.885)	0.506	0.08	0.400 ± 0.078	0.320 ± 0.080	−0.08	0.775 (0.659, 0.891)	0.530	0.082	0.381 ± 0.065	0.310 ± 0.075	−0.071	0.782 (0.669, 0.896)	0.498	0.075
**Healthy Group**	**No vibration (*N* = 1273 strides)**						**Stance vibration (*N* = 411 strides)**						**Swing vibration (*N* = 394 strides)0**					
	**Mean ± SD** **Vicon**	**Mean ± SD** **NUSHU**	**Mean difference**	**ICC** **(95% CI)**	**CCC**	**MAE**	**Mean ± SD** **Vicon**	**Mean ± SD** **NUSHU**	**Mean difference**	**ICC** **(95% CI)**	**CCC**	**MAE**	**Mean ± SD** **Vicon**	**Mean ± SD** **NUSHU**	**Mean difference**	**ICC** **(95% CI)**	**CCC**	**MAE**
Stride Length (m)	1.389 ± 0.153	1.334 ± 0.157	−0.055	0.875 (0.798, 0.952)	0.842	0.072	1.361 ± 0.144	1.317 ± 0.147	−0.044	0.876 (0.800, 0.952)	0.841	0.065	1.401 ± 0.158	1.350 ± 0.166	−0.051	0.895 (0.829, 0.961)	0.870	0.068
Stride time (s)	1.113 ± 0.085	1.115 ± 0.087	0.002	0.814 (0.707, 0.921)	0.959	0.017	1.123 ± 0.068	1.124 ± 0.069	0	0.834 (0.737, 0.932)	0.929	0.017	1.108 ± 0.085	1.112 ± 0.086	0.004	0.866 (0.785, 0.948)	0.963	0.015
Velocity (m/s)	1.260 ± 0.208	1.209 ± 0.220	−0.051	0.880 (0.806, 0.954)	0.924	0.067	1.218 ± 0.174	1.180 ± 0.182	−0.038	0.905 (0.845, 0.965)	0.908	0.061	1.277 ± 0.217	1.229 ± 0.233	−0.048	0.922 (0.871, 0.972)	0.938	0.065
Double support time (s)	0.399 ± 0.059	0.326 ± 0.051	−0.073	0.735 (0.597, 0.874)	0.419	0.073	0.406 ± 0.053	0.330 ± 0.044	−0.076	0.762 (0.633, 0.891)	0.315	0.077	0.389 ± 0.063	0.316 ± 0.051	−0.073	0.796 (0.681, 0.911)	0.415	0.073

SD = Standard deviation, ICC = Intraclass correlation coefficient, CI = Confidence interval, CCC = Concordance correlation coefficient, MAE = Mean absolute error.

## Data Availability

Summary data for this study are available from the corresponding author upon reasonable request.

## Supplementary Materials

The following supporting information can be downloaded at https://www.mdpi.com/article/10.3390/s26144446/s1. Detailed description of synchronisation procedure.

## Abbreviations

The following abbreviations are used in this manuscript:

CI	Confidence Interval
ICC	Intraclass Correlation Coefficient
IMU	Inertial Measurement Unit
MAE	Mean Absolute Error
MCID	Minimal Clinically Important Difference
MDS-UPDRS	Movement Disorder Society-Unified Parkinson’s Disease Rating Scale
MoCA	Montreal Cognitive Assessment
PD	Parkinson’s disease
SD	Standard Deviation

## References

[1] Burtscher J., Moraud E.M., Malatesta D., Millet G.P., Bally J.F., Patoz A. (2024). Exercise and gait/movement analyses in treatment and diagnosis of Parkinson’s Disease. Ageing Res. Rev..

[2] Mirelman A., Bonato P., Camicioli R., Ellis T.D., Giladi N., Hamilton J.L., Hass C.J., Hausdorff J.M., Pelosin E., Almeida Q.J. (2019). Gait impairments in Parkinson’s disease. Lancet Neurol..

[3] Zanardi A.P.J., da Silva E.S., Costa R.R., Passos-Monteiro E., dos Santos I.O., Kruel L.F.M., Peyré-Tartaruga L.A. (2021). Gait parameters of Parkinson’s disease compared with healthy controls: A systematic review and meta-analysis. Sci. Rep..

[4] Cramer L.A., Wimmer M.A., Malloy P., Joan A.O.K., Knowlton C.B., Ferrigno C. (2022). Validity and Reliability of the Insole3 Instrumented Shoe Insole for Ground Reaction Force Measurement during Walking and Running. Sensors.

[5] Pau M., Corona F., Pili R., Casula C., Guicciardi M., Cossu G., Murgia M. (2018). Quantitative assessment of gait parameters in people with Parkinson’s disease in laboratory and clinical setting: Are the measures interchangeable?. Neurol. Int..

[6] Scataglini S., Abts E., Van Bocxlaer C., Van den Bussche M., Meletani S., Truijen S. (2024). Accuracy, Validity, and Reliability of Markerless Camera-Based 3D Motion Capture Systems versus Marker-Based 3D Motion Capture Systems in Gait Analysis: A Systematic Review and Meta-Analysis. Sensors.

[7] Smith M.D., Brazier D.E., Henderson E.J. (2021). Current Perspectives on the Assessment and Management of Gait Disorders in Parkinson’s Disease. Neuropsychiatr. Dis. Treat..

[8] Rastegari E., Marmelat V., Najjar L., Bastola D., Ali H.H. Using gait parameters to recognize various stages of Parkinson’s disease. Proceedings of the 2017 IEEE International Conference on Bioinformatics and Biomedicine (BIBM).

[9] Kirk C., Packer E., Polhemus A., MacLean M.K., Bailey H., Kluge F., Gaßner H., Rochester L., Del Din S., Yarnall A.J. (2025). A systematic review of real-world gait-related digital mobility outcomes in Parkinson’s disease. npj Digit. Med..

[10] Maetzler W., Domingos J., Srulijes K., Ferreira J.J., Bloem B.R. (2013). Quantitative wearable sensors for objective assessment of Parkinson’s disease. Mov. Disord..

[11] Kirk C., Rehman R.Z.U., Galna B., Ranciati S., Packer E., Ireson N., Lanfranchi V., Mazzà C., Alcock L., Rochester L. (2026). Toward an understanding of real-world mobility in Parkinson’s: Insights from enhanced contextualisation using GPS-derived location and data-driven modeling of walking speed. Front. Aging Neurosci..

[12] Nonnekes J., Snijders A.H., Nutt J.G., Deuschl G., Giladi N., Bloem B.R. (2015). Freezing of gait: A practical approach to management. Lancet Neurol..

[13] Huang P., Mostovov A., Cohen R., Cadilhac C., Pionnier R. (2025). Comparison of feetme insoles with a motion capture system coupled to force plates for assessing gait and posture. Sci. Rep..

[14] Jaeschke R., Singer J., Guyatt G.H. (1989). Measurement of health status: Ascertaining the minimal clinically important difference. Control. Clin. Trials.

[15] Lee M., Youm C., Jeon J., Cheon S.-M., Park H. (2018). Validity of shoe-type inertial measurement units for Parkinson’s disease patients during treadmill walking. J. Neuroeng. Rehabil..

[16] Pergolini A., Bowman T., Lencioni T., Marzegan A., Meloni M., Carrozza M.C., Trigili E., Vitiello N., Cattaneo D., Crea S. (2024). Assessment of Sensorized Insoles in Balance and Gait in Individuals With Parkinson’s Disease. IEEE Trans. Neural Syst. Rehabil. Eng..

[17] Esser P., Dawes H., Collett J., Feltham M.G., Howells K. (2012). Validity and inter-rater reliability of inertial gait measurements in Parkinson’s disease: A pilot study. J. Neurosci. Methods.

[18] Morris R., Stuart S., McBarron G., Fino P.C., Mancini M., Curtze C. (2019). Validity of Mobility Lab (version 2) for gait assessment in young adults, older adults and Parkinson’s disease. Physiol. Meas..

[19] Parati M., Gallotta M., Muletti M., Pirola A., Bellafà A., De Maria B., Ferrante S. (2022). Validation of Pressure-Sensing Insoles in Patients with Parkinson’s Disease during Overground Walking in Single and Cognitive Dual-Task Conditions. Sensors.

[20] Wu J., Kuruvithadam K., Schaer A., Stoneham R., Chatzipirpiridis G., Easthope C.A., Barry G., Martin J., Pané S., Nelson B.J. (2021). An Intelligent In-Shoe System for Gait Monitoring and Analysis with Optimized Sampling and Real-Time Visualization Capabilities. Sensors.

[21] Redgrave P., Rodriguez M., Smith Y., Rodriguez-Oroz M.C., Lehericy S., Bergman H., Agid Y., DeLong M.R., Obeso J.A. (2010). Goal-directed and habitual control in the basal ganglia: Implications for Parkinson’s disease. Nat. Rev. Neurosci..

[22] El-Tamawy M.S., Darwish M.H., Khallaf M.E. (2012). Effects of augmented proprioceptive cues on the parameters of gait of individuals with Parkinson’s disease. Ann. Indian. Acad. Neurol..

[23] Phuenpathom W., Panyakaew P., Vateekul P., Surangsrirat D., Hiransuthikul A., Bhidayasiri R. (2022). Vibratory and plantar pressure stimulation: Steps to improve freezing of gait in Parkinson’s disease. Park. Relat. Disord..

[24] Phuenpathom W., Panyakaew P., Vateekul P., Surangsrirat D., Bhidayasiri R. (2024). Residual effects of combined vibratory and plantar stimulation while seated influences plantar pressure and spatiotemporal gait measures in individuals with Parkinson’s disease exhibiting freezing of gait. Front. Aging Neurosci..

[25] Chinmilli P., Redkar S., Zhang W., Sugar T. (2017). A review on wearable inertial tracking based human gait analysis and control strategies of lower-limb exoskeletons. Int. Robot. Autom. J..

[26] Karlawish J., Cary M., Moelter S.T., Siderowf A., Sullo E., Xie S., Weintraub D. (2013). Cognitive impairment and PD patients’ capacity to consent to research. Neurology.

[27] Bonett D.G. (2002). Sample size requirements for estimating intraclass correlations with desired precision. Stat. Med..

[28] Walter S.D., Eliasziw M., Donner A. (1998). Sample size and optimal designs for reliability studies. Stat. Med..

[29] Wu J., Maurenbrecher H., Schaer A., Becsek B., Easthope C.A., Chatzipirpiridis G., Ergeneman O., Pané S., Nelson B.J. (2022). Human gait-labeling uncertainty and a hybrid model for gait segmentation. Front. Neurosci..

[30] Portney L.G., Watkins M.P. (2009). Foundations of Clinical Research: Applications to Practice.

[31] Bland J.M., Altman D.G. (1986). Statistical methods for assessing agreement between two methods of clinical measurement. Lancet.

[32] Baudendistel S.T., Haussler A.M., Rawson K.S., Earhart G.M. (2024). Minimal clinically important differences of spatiotemporal gait variables in Parkinson disease. Gait Posture.

[33] Del Din S., Elshehabi M., Galna B., Hobert M.A., Warmerdam E., Suenkel U., Brockmann K., Metzger F., Hansen C., Berg D. (2019). Gait analysis with wearables predicts conversion to parkinson disease. Ann. Neurol..

[34] Agurto C., Heisig S., Abrami A., Ho B.K., Caggiano V. (2021). Parkinson’s disease medication state and severity assessment based on coordination during walking. PLoS ONE.

[35] Verghese J., Holtzer R., Lipton R.B., Wang C. (2009). Quantitative Gait Markers and Incident Fall Risk in Older Adults. J. Gerontol. Ser. A.

[36] Byun S., Han J.W., Kim T.H., Kim K., Kim T.H., Park J.Y., Suh S.W., Seo J.Y., So Y., Lee K.H. (2018). Gait Variability Can Predict the Risk of Cognitive Decline in Cognitively Normal Older People. Dement. Geriatr. Cogn. Disord..

[37] Weir J.P. (2005). Quantifying Test-Retest Reliability Using the Intraclass Correlation Coefficient and the Sem. J. Strength Cond. Res..

[38] Atkinson G., Nevill A.M. (1998). Statistical Methods for Assessing Measurement Error (Reliability) in Variables Relevant to Sports Medicine. Sports Med..

[39] Studenski S., Perera S., Patel K., Rosano C., Faulkner K., Inzitari M., Brach J., Chandler J., Cawthon P., Connor E.B. (2011). Gait speed and survival in older adults. JAMA.

[40] Fritz S., Lusardi M. (2009). White paper: “walking speed: The sixth vital sign”. J. Geriatr. Phys. Ther..

