# Supplementary material for: A Smart Shoe System for Gait Analysis and Remote Monitoring in Parkinson’s Disease—A Validation Study
Source: Sensors (Basel). 2026 Jul 13;26(14):4446. doi: 10.3390/s26144446 (PMC13417132; doi:10.3390/s26144446)
Supplement: Supplementary file 1 [file sensors-26-04446-s001.zip › sensors-4309200-supplementary.pdf]

Supplementary Material

NUSHU-Vicon Synchronisation

Signal Sources and Parameters

The NUSHU system recorded tri-axial linear acceleration and angular velocity at a sampling frequency of 100 Hz, with data expressed relative to the start of each recording. The Vicon system recorded corresponding signals from inertial sensors attached to each shoe at a higher sampling frequency of 1125 Hz.

At the start of each measurement, the NUSHU system generated two brief vibration pulses (200 ms each) using the embedded motors. The motor activation signal (ON/OFF) was recorded alongside the sensor data and used as a reference for synchronisation. These vibration events were also detected by the linear accelerometers of the Vicon IMUs. Synchronisation was performed separately for the left and right sides.

Synchronisation Strategies

Three strategies were used to estimate the temporal offset (Δt) between the NUSHU and Vicon systems: (1) cross-correlation of linear acceleration signals, (2) cross-correlation of angular velocity signals, and (3) a vibration-based event detection approach.

The first two approaches were based on cross-correlation of inertial signals, including motion, while the third used changes in signal energy associated with vibration events before the start of ambulation, solely (i.e., excluding motion). These approaches are described in detail in the following subsections.

Cross-correlation of linear acceleration signals and angular velocity signals

The first two approaches estimated the temporal offset by identifying shared inertial patterns in signals recorded by both systems using cross-correlation. This method was applied to both linear acceleration and angular velocity signals using the same processing steps.

Because the Vicon IMU data were recorded at a higher sampling frequency than the NUSHU data, the Vicon signals were resampled to match the NUSHU sampling rate, with appropriate low-pass filtering applied to avoid aliasing. To minimise the influence of sensor orientation differences between systems, the magnitude of each tri-axial signal was calculated, resulting in a single time series for each signal. These signals were then standardised to enable comparison.

To reduce computational load whilst ensuring that at least one dynamically rich event (e.g., a step) was present, the analysis was limited to the initial segment of each recording (the first 10 seconds). Cross-correlation was then performed between the processed NUSHU and Vicon signals, and the time lag corresponding to the highest correlation was identified as the temporal offset (Δt). This offset was subsequently used to align the NUSHU data with the Vicon time base. The procedure was performed independently for the left and right sides.

Vibration-based event detection (Signal Energy Strategy)

The third approach utilised a consistent event embedded within each NUSHU recording. At the start of each trial, the NUSHU system generated two brief vibration pulses, which were recorded as a binary motor signal (ON/OFF) and simultaneously detected as mechanical vibrations in the Vicon IMU accelerometer data. Aligning these corresponding events between the two systems enabled estimation of the temporal offset.

Two characteristic points of the vibration signal were considered for alignment: the midpoint of the vibration event or the second falling edge. This dual approach improved robustness in cases where disturbances (e.g.

additional movements) or excessive noise affected part of the signal. The most appropriate event marker was selected through visual inspection of each recording.

This method involved three main steps: (1) identifying the vibration event in the NUSHU data, (2) detecting the corresponding event in the Vicon data, and (3) calculating the time difference between the two events to determine the synchronisation offset. As this approach relied on discrete event timing, signal resampling was not required.

Extraction of the NUSHU Vibration Event Time

The vibration event was identified from the binary motor signal recorded by the NUSHU system. Rising and falling edges were detected to determine the onset and offset of the two initial vibration pulses.

The event time for NUSHU was defined using one of two approaches: either the midpoint between the first rising edge and the second falling edge of the pulses, or the second falling edge alone. The selected definition was used to represent the timing of the vibration event.

Extraction of the Vicon vibration event time

To identify the vibration event in the Vicon accelerometer data, analysis was restricted to a short time window at the beginning of each recording (approximately 4 seconds), corresponding to the stationary vibration period. This gave us control over the influence of movement-related artefacts.

Within this window, the magnitude of the tri-axial accelerometer signal was calculated to minimise the effect of sensor orientation. A local energy signal was then derived from this magnitude signal using a moving window, and subsequently standardised. A threshold was applied to detect periods of vibration activity.

The times corresponding to the onset and offset of the detected vibration were identified. The Vicon vibration event time was defined as either the midpoint between these events or the final offset, depending on the selected implementation.

Motor transient compensation

A short delay exists between the command to activate the NUSHU vibration motor and the appearance of the resulting mechanical vibration in the accelerometer signal. Based on prior characterisation, this delay was estimated at 50 ms and assumed to be similar for both activation and deactivation.

To account for this systematic delay, a correction factor ($\delta t = 50$ ms) was added to the estimated synchronisation offset. The temporal offset for the vibration-based approach was calculated as the difference between the NUSHU and Vicon event times, after applying the correction. This procedure was performed independently for the left and right sides.

Manual Selection of the Synchronisation Time

Each approach produced candidate synchronisation offsets for each side (three values per side). These offsets were manually evaluated by visually inspecting their effect on signal alignment. Specifically, the NUSHU vibration signal was compared in its original and time-shifted forms against the Vicon IMU acceleration magnitude. As the vibration events were clearly identifiable in the Vicon signal, this allowed selection of the offset that provided the best alignment.

This process helped mitigate potential limitations of individual methods, such as sensitivity to motion artefacts in the vibration-based approach or dependence on signal variability in the cross-correlation approach, ensuring robust synchronisation.

Application of Synchronisation

Once the optimal synchronisation offset (Δt) was selected for each side, it was applied to the NUSHU timestamps to align the signals with the Vicon system. Following temporal alignment, the first heel strike event identified by both systems and showing close temporal agreement was defined as time zero for that trial.
